# Interactivity and Reward-Related Neural Activation during a Serious Videogame

**DOI:** 10.1371/journal.pone.0033909

**Published:** 2012-03-19

**Authors:** Steven W. Cole, Daniel J. Yoo, Brian Knutson

**Affiliations:** 1 HopeLab Foundation, Redwood City, California, United States of America; 2 Department of Psychology, Stanford University, Stanford, California, United States of America; Sanford-Burnham Medical Research Institute, United States of America

## Abstract

This study sought to determine whether playing a “serious” interactive digital game (IDG) – the *Re-Mission* videogame for cancer patients – activates mesolimbic neural circuits associated with incentive motivation, and if so, whether such effects stem from the participatory aspects of interactive gameplay, or from the complex sensory/perceptual engagement generated by its dynamic event-stream. Healthy undergraduates were randomized to groups in which they were scanned with functional magnetic resonance imaging (FMRI) as they either actively played *Re-Mission* or as they passively observed a gameplay audio-visual stream generated by a yoked active group subject. Onset of interactive game play robustly activated mesolimbic projection regions including the caudate nucleus and nucleus accumbens, as well as a subregion of the parahippocampal gyrus. During interactive gameplay, subjects showed extended activation of the thalamus, anterior insula, putamen, and motor-related regions, accompanied by decreased activation in parietal and medial prefrontal cortex. Offset of interactive gameplay activated the anterior insula and anterior cingulate. Between-group comparisons of within-subject contrasts confirmed that mesolimbic activation was significantly more pronounced in the active playgroup than in the passive exposure control group. Individual difference analyses also found the magnitude of parahippocampal activation following gameplay onset to correlate with positive attitudes toward chemotherapy assessed both at the end of the scanning session and at an unannounced one-month follow-up. These findings suggest that IDG-induced activation of reward-related mesolimbic neural circuits stems primarily from participatory engagement in gameplay (interactivity), rather than from the effects of vivid and dynamic sensory stimulation.

## Introduction

Play represents a distinctive behavioral repertoire that is both highly rewarding and evolutionarily conserved [1]. “Serious games” seek to promote positive changes in attitudes and behavior by leveraging fundamental neural processes engaged by play [2]–[6]. Despite burgeoning interest in serious games and data showing that they can provide powerful tools for altering attitudes and behavior [3]–[5], [7], [8], the psychological mechanisms of their effects remain poorly defined. Several studies have documented activation of mesolimbic circuits associated with reward anticipation and incentive motivation as people play “non-serious” entertainment-oriented interactive digital games (IDGs; colloquially known as “videogames”) [9]–[12]. Activation of brain motivational systems has been hypothesized to mediate the positive behavioral impact of serious IDGs (i.e., those explicitly designed to alter real-world attitudes and behavior) [3], [6], [13], but it is not clear which specific aspects of the IDG play experience engage those motivational processes. Identification of the key motivation-engaging features of IDGs would significantly enhance our ability to rationally engineer play experiences that maximally influence attitudes and behavior.

One theoretical perspective suggests that the distinctive motivational impact of IDG play is a consequence of processing the complex, dynamic, and multi-modal sensory stream of events generated by interactive games [3], [13], [14]. This account likens the IDG experience to other vivid, dynamic, emotionally engaging, multi-modal perceptual stimuli (e.g., audio-visual entertainment, stories, etc.) that have been found to enhance motivation, learning, and memory [14]. An alternative perspective suggests that the distinctive neural responses to IDG play stem not from the mere observation of a dynamic event stream, but rather rom the player's personal participation in shaping that dynamic event stream [3], [13]. Under this hypothesis, the neural responses to IDG play differ qualitatively from those evoked by other highly vivid, dynamic, and emotionally engaging stimuli that do not involve the behavioral participation of the observer (e.g., non-interactive audio-visual entertainment).

In the present study, we sought to evaluate these alternative hypotheses in the context of a serious IDG that is already known to significantly impact behavior. The *Re-Mission* videogame was developed as a rationally targeted IDG behavioral intervention to improve health outcomes in adolescents and young adults currently undergoing treatment for cancer [7], [8], [15]. A randomized controlled trial of *Re-Mission* in 374 adolescent and young adult cancer patients showed that the game significantly enhanced several targeted psychological and behavioral outcomes, including knowledge about cancer, self-efficacy to overcome the disease, and adherence to self-administered oral chemotherapy regimens [8]. The current study seeks to understand *Re-Mission*'s general effects on the activation of reward-related neural circuits previously hypothesized to contribute to IDG-induced behavior change. After verifying that, (1) playing *Re-Mission* activates the same mesolimbic incentive motivation circuits previously found to be engaged by entertainment-targeted (i.e., non-serious) IDGs (e.g., the nucleus accumbens and broader mesolimbic dopamine projection areas) [9]–[12], this study tested the primary hypothesis that (2) activation of mesolimbic circuits is driven primarily by the interactive or participatory nature of gameplay, as opposed to the vivid, dynamic sensory stream it generates (i.e., different neural responses are generated by exposure to an identical non-interactive audio-visual information stream in a yoked passive control condition). Additional exploratory analyses also assessed the possibility that (3) the magnitude of gameplay-induced neural activation is associated with post-play differences in attitudes toward cancer chemotherapy – a key psychological target of *Re-Mission* as a serious IDG [7], [8].

## Results

**Figure 1:**
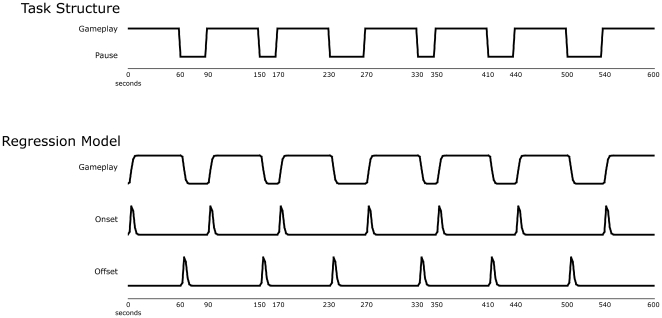
Task structure and regressors of interest. Play vs. pause structure (top) and FMRI signal regressors capturing general effects of Gameplay vs rest, Gameplay onset, and Gameplay offset.

Fifty-seven healthy undergraduates were each randomized to one of two experimental groups and scanned with functional magnetic resonance imaging (FMRI) as they either actively played the cancer-related *Re-Mission* videogame for seven bouts of 60 sec separated by six rest pauses of variable 10–30 sec duration (“active play” group; Supporting Information Video 1) or passively observed a gameplay audio-visual stream generated by a yoked active group player (“passive exposure” group). Standard contrast-based analyses of T2* data (spiral in/out pulse sequence, repetition time = 2 sec, echo time = 40 ms, flip = 90 degrees) examined within-subject changes in neural activity during periods of gameplay versus rest (to assess general neural correlates of gameplay), as well as 2 sec intervals immediately following gameplay onset (to assess transient activation of incentive motivation-related neural structures, as previously observed [16], [17]), and immediately following gameplay offset (to determine whether offset responses mimic 2 sec onset responses and might thus reflect responses to transition or novelty [18], or whether they reflect motivational dynamics, in which case one might expect activation in distinct neural territories associated with negative motivational responses [19], [20]). Figure 1 shows within-subject longitudinal regressors capturing each effect. The extent to which interactivity enhanced each of those within-subject contrast dynamics was assessed in between-group comparisons.

### Interactive gameplay versus rest

**Figure 2:**
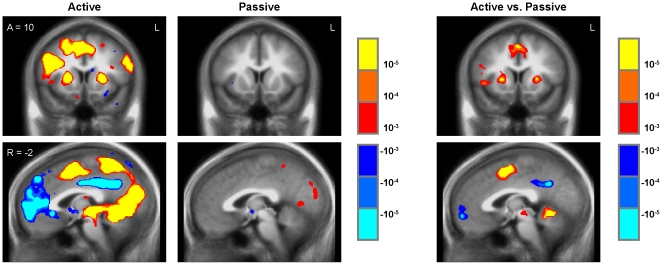
Gameplay versus rest. Play vs. rest regressor for Active Play and Passive Exposure groups (left panels) and Active vs. Passive group comparison (right panels). All threshold p<.001, uncorrected.

Initial whole-brain analyses examined the general correlates of interactive gameplay versus rest in each experimental group. Within the active play group, the play versus rest contrast was associated with wide-spread activation changes in diverse brain regions (Figure 2; Table S1a), including expected increases in activation within sensory regions (i.e., primary visual cortex) and motor regions (i.e., primary motor cortex), as well as thalamus, anterior insula, and putamen (i.e., included within the extended culmen focus cluster in Table S1a). Unexpectedly, the active play versus rest contrast was also negatively associated with activation in medial prefrontal cortex, posterior cingulate cortex, and subregions of the medial temporal lobe (i.e., included within the extended middle temporal gyrus clusters in Table S1a). In the passive exposure group, viewing the yoked audio-visual stream generated by interactive gameplay was positively associated with activation in sensory regions (i.e., visual cortex and precuneus) but not motor regions (Figure 2; Table S1b). Between-group comparison of the active play group versus the passive exposure group for the play versus rest contrast revealed interactivity-related increases in activation within regions associated with motor function (i.e., dorsolateral prefrontal cortex, anterior cingulate, supplementary motor area, motor cortex) as well as anterior insula, putamen, and thalamus (i.e., included within the extended superior frontal gyrus cluster in Table S1c), but decreased activation in medial prefrontal cortex, subterritories of the medial temporal lobe, and parietal cortex (Figure 2; Table S1c).

### Gameplay onset

**Figure 3:**
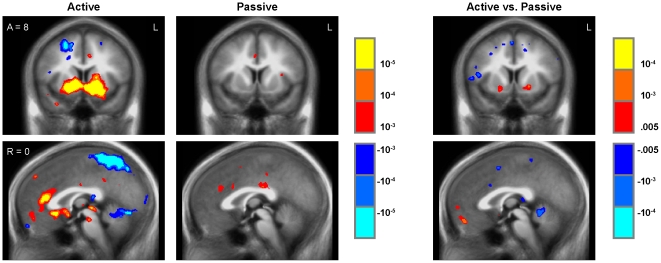
Gameplay onset. Play onset regressor for Active Play and Passive Exposure groups (left and middle panels, threshold p<.001, uncorrected, in whole-brain analysis) and Active vs. Passive group comparison (right panels, threshold p<.005, VOI-based).

Because reward-related neural activity often peaks transiently in response to the appearance or anticipation of motivationally-relevant stimuli [21], [22], primary analyses assessed effects of interactive gameplay on reward-related mesolimbic activation during the first 2 sec following gameplay onset. As hypothesized, game onset in the active play group was associated with increased neural activity in the striatum (including the nucleus accumbens, caudate, and putamen), anterior cingulate, and posterior insula within the active play group (Figure 3). Whole-brain analyses identified a diverse array of additional regional effects, including activation in subregions of parahippocampal gyrus (e.g., Right/Anterior/Superior RAS coordinates = 26, −15, −14) (Figure 3; Table S2a). Initiation of interactive gameplay significantly decreased activity in the dorsolateral prefrontal cortex and parietal cortex (Figure 3). In the passive exposure group, initiation of the same complex audiovisual stimulus stream was associated with increased activity in the anterior cingulate, anterior insula, and parahippocampal regions, and decreased activity in the right precuneus, inferior temporal gyrus, and middle temporal gyrus (Figure 3; Table S2b). Between-group comparison of neural responses to game onset using an a priori volume-of-interest (VOI/region of interest) analysis confirmed that interactive gameplay induced greater increases in the bilateral nucleus accumbens and bilateral ventral putamen activation (individually p<.025, corresponding to Bonferroni-corrected p<.05; Table 1). Whole-brain analyses of between-group differences also identified increased activation in the left parahippocampal gyrus (at the a priori VOI threshold) and decreased activation in the right anterior insula, right dorsolateral prefrontal cortex, and parietal cortex in the active play group relative to the passive exposure group (p<.001; Figure 3; Table S2c).

**Table 1:** Game onset a priori volume-of-interest (VOI) analysis.

Volume of Interest	Coordinates of 6 mm diameter sphere	Active	Passive	t-statistic	Uncorrectedp-value
Bilateral NAcc*	Mean:	0.2340	0.0600	2.67	0.0101
	SD:	*0.2015*	*0.1924*		
Left NAcc**	(−11, 11, −2)	0.2420	0.0675	2.59	0.0125
		*0.2154*	*0.1694*		
Right NAcc	(11, 11, −2)	0.2260	0.0525	2.27	0.0275
		*0.2255*	*0.2658*		
Bilateral Putamen*	Mean:	0.3010	0.0507	3.58	0.0007
	SD:	*0.2153*	*0.2090*		
Left Putamen**	(−17, 12, 0)	0.3191	0.0802	3.22	0.0022
		*0.2283*	*0.2235*		
Right Putamen**	(17, 12, 0)	0.2829	0.0211	3.41	0.0012
		*0.2381*	*0.2219*		

* Primary analysis significant at p<.025 uncorrected, p<.05 Bonferroni-corrected.

** Secondary analysis significant at p<.0125 uncorrected, p<.05 Bonferroni-corrected.

NAcc = nucleus accumbens.

### Gameplay offset

**Figure 4:**
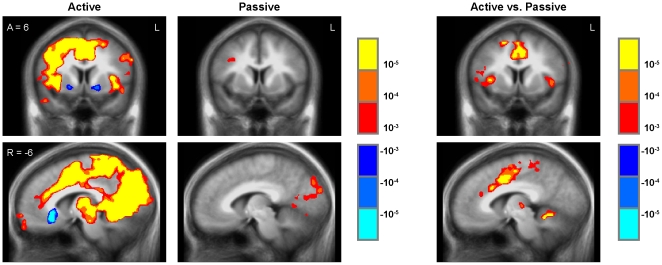
Gameplay offset. Play offset regressor for Active Play and Passive Exposure groups (left panels) and Active vs. Passive group comparison (right panels). All threshold p<.001, uncorrected, in whole-brain analysis.

To determine whether the transient neural activations associated with gameplay onset stemmed primarily from changes in game status or event boundaries [18], rather than reward-related processes, we also examined 2 sec responses to gameplay offset. Under the “mere change” hypothesis, gameplay offset should induce changes in neural activity similar to those associated with gameplay onset, whereas the “reward” hypothesis would predict distinct patters of offset-related activation in neural structures implicated in negative reactions or interruption [19], [20]. Consistent with the latter account, the 2-sec period following gameplay offset was associated in the active play group with distinct but diverse regional effects including activation of the anterior insula, anterior cingulate (i.e., included within the extended middle occipital gyrus cluster in Table S3a), posterior cingulate, dorsolateral prefrontal cortex, premotor cortex, primary motor cortex, parietal cortex, and precuneus, as well as decreased activity in the orbitofrontal cortex, caudate, putamen, and left inferior parietal lobule (Figure 4; Table S3a). In the passive exposure group, the same gameplay offset regressor was associated with increased activation in the inferior frontal gyrus, superior temporal gyrus, precentral gyrus, lingual gyrus, posterior cingulate, precuneus, and primary visual cortex, as well as decreased activity in the middle frontal gyrus, postcentral gyrus, middle insula, and parahippocampal gyrus (Figure 4; Table S3b). Statistical comparison of gameplay offset responses in the active play versus passive exposure groups indicated significant increased activity in the anterior insula, anterior cingulate, inferior frontal gyrus, supplementary motor area, primary motor cortex, thalamus, and culmen (Figure 4; Table S3c). Thus, gameplay offset recruited a set of neural structures distinct from those activated by game play onset and generally associated with anticipation of loss and motor conflict.

### Player-generated in-game events

The activation of mesolimbic projection regions during interactive gameplay might stem predominately from player-generated in-game “success events” such as firing a chemoblaster shot or killing an enemy cancer cell, rather than the more general complex of interactivity-related processes (which include a variety of other psychological processes such as planning, navigation, threat/obstacle evasion, etc.). Analysis of regressors tracking enemy cancer cells killed and chemoblaster shots fired revealed scattered patterns of correlated activation within the active play group (Table S4, S5), but no significant association with the reward-related mesolimbic regions targeted in this study.

### Attitudes toward chemotherapy

**Figure 5:**
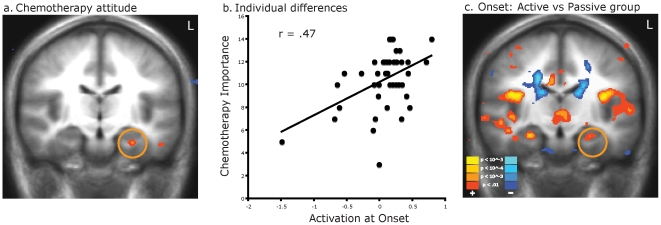
In-game neural response associated with post-gameplay attitude toward chemotherapy. (a) Individual differences in attitudes toward chemotherapy measured immediately after the interactive gameplay session in the active play group were associated with increased activation in several brain regions including a subregion of the left parahippocampal cortex (threshold p<.001, uncorrected, in whole-brain analysis). (b) The strength of this relationship was quantified by correlating individual average response following gameplay onset (measured at empirical peak response 6 sec after gameplay onset in a 6 mm VOI centered on the empirical response focus) with post-play attitudes toward chemotherapy. (c) The same general region of left parahippocampal gyrus also showed significantly greater activation in the active play group following gameplay onset than in the passive observation group (threshold p<.001, uncorrected).

To determine whether any of the neural responses associated with the onset of interactive gameplay might potentially relate to post-play attitudes toward chemotherapy (a primary target of *Re-Mission* as a serious game [7], [8]), we conducted individual difference analyses within the active play group relating the magnitude of peak neural response to gameplay onset (empirically determined to occur 6 sec after gameplay onset) to attitudes toward chemotherapy measured immediately after the FMRI scanning session and at an unannounced follow-up one month later. The magnitude of response in striatal regions showed no significant association with the positivity of post-play or follow-up attitudes. However, immediate post-play endorsement of the importance of chemotherapy correlated with game onset-induced activation in the bilateral anterior cingulate, bilateral mesial prefrontal cortex, and a subregion of the left parahippocampal gyrus (RAS = −26,−15,−19) within the active play group (Figure 5; Table S6). The attitude-related left parahippocamal activation focus partially overlapped with a left parahippocampal cortex territory that showed greater play onset-associated activation in the active play group relative to the passive exposure group (Figure 5c). To graphically portray the association between attitudes and left parahippocampal activation following gameplay onset, we plotted the relationship between individual differences in average peak activation (empirically determined to fall at 6 sec after play onset) in a 6 mm diameter VOI centered on the attitude-related response focus (Figure 5a) and chemotherapy-related attitudes. Results showed a substantial linear association (Pearson r(55) = .47; outlier-robust Spearman r(55) = .38 (Figure 5b). Perceived importance of chemotherapy assessed one month after the gameplay session was also associated with greater game onset response in the left inferior frontal gyrus and a subregion of the left parahippocampal gyrus adjacent to that associated with attitudes measured immediately post-play (Z = 3.49; RAS = −33,−7,−20) (Table S6b). Peak activation within a 6 mm diameter volume centered on this focus also showed a linear association with perceived importance of chemotherapy at one month follow-up (Pearson r(38) = .45; Spearman r(38) = .34).

## Discussion

This study sought to determine which aspect of a “serious game” play experience drives recruitment of mesolimbic projection areas associated with reward motivation [9]–[12]. The *Re-Mission* videogame has previously been found to enhance psychological and medical treatment-related behavioral outcomes in young people being treated for cancer [7], [8], and the present data show that playing *Re-Mission* can markedly activate neural circuits implicated in reward (i.e., caudate, putamen, and nucleus accumbens). These data also identify the participatory nature of interactive gameplay as a key driver of those neural responses. Temporal analyses of transition between pause conditions and active gameplay confirmed that ventral striatal activation was not driven solely by state transition-induced arousal (as might be hypothesized under theories interpreting mesolimbic dopamine activity in terms of novelty [23]). Subjects in a passive exposure group experienced the same novel, vivid, dynamic, and emotionally involving stimulus streams as did those actively playing *Re-Mission*, but showed markedly less recruitment of mesolimbic structures implicated in reward processing. Thus, the present findings are consistent with previous analyses of cancer-related self-efficacy [8] in suggesting that personal involvement and agency may represent key psychological drivers of *Re-Mission's* impact on behavior. While the highly vivid and dynamic event streams generated during IDG play do activate sensory neural structures, player involvement in shaping the event stream (i.e., interactivity) was required to substantially engage motivation-related brain circuits in response to *Re-Mission* gameplay. Although the present study focused on the impact of interactivity in the context of a serious game, interactivity likely contributes to reward-related neural activation during non-serious entertainment-oriented IDGs as well [9]–[12]. What this study reveals about serious IDGs is that participatory interaction plays a key role in engaging reward-related neural processes previously hypothesized to mediate serious games' distinctive impact on “out-of-game” attitudes and behaviors (i.e., their potentially distinctive active ingredient relative to other attitude/behavior-change interventions) [3], [6], [13].

In addition to activating the reward-related ventral striatum, the onset of interactive gameplay also increased activation in several other relevant regions. Of particular interest were subregions of the parahippocampal cortex falling at the interface between CA1 and the subiculum, which showed increased activation in response to the onset of interactive gameplay. In active play subjects, the quantitative magnitude of game onset-induced activation in regions of the left parahippocampal cortex positively correlated with the intensity of positive attitudes toward cancer chemotherapy assessed immediately following gameplay, as well as chemotherapy-related attitudes assessed one month later in an unannounced follow-up. In the passive exposure group, however, the quantitative magnitude of parahippocampal activation showed no significant correlation with post-play chemotherapy-related attitudes. We did not assess baseline chemotherapy-related attitudes prior to gameplay (by design, in order to avoid report-induced attitude anchoring), so it is not clear whether gameplay-induced neural responses in the left parahippocampal cortex are associated with gameplay-induced attitude change, or whether they might instead relate to pre-existing individual differences in attitude. *Re-Mission* has been shown to alter patients' cancer-related attitudes and behavior in previous studies [7], [8], but future analyses assessing pre-play attitudes will be required to more clearly define the role of play-induced parahippocampal activation on attitude change. Given the key role of the hippocampus in learning and memory, it is conceivable that IDG-induced activation in this region might potentially play a role in translating short-term play experiences into long-term effects on attitudes or behavior. However, direct measures of learning, memory, and motivation not available in this study will be required to test that hypothesis. Ultimately, studies will need to simultaneously contrast the effects of serious and non-serious IDGs on neural system engagement “in-game,” direct measures of play-induced learning and motivation, and “out-of-game” changes in attitude and behavior, in order to fully define the neural mechanisms by which serious IDGs exert their distinctive effects on behavior. The present results suggest that reward-related mesolimbic regions and the left parahippocampal cortex might serve as “candidate regions of interest” for such future studies.

Another unexpected finding was that extended periods of active IDG play were associated with reduced activity in some regions, including the medial prefrontal cortex, striatum, and medial temporal lobe (Figure 2). Future studies will be required to replicate these findings and determine their psychological basis, but such dynamics would be consistent with the hypothesis that playing IDGs alters the cognitive pathways through which information is processed [3], [6], [13]–[15]. For example, the observed reductions in medial prefrontal activity during extended periods of gameplay would be consistent with hypotheses that gameplay (1) inhibits controlled information processing and preferentially activates more automatic subcortical systems that focus on immediate goals [24], [25], (2) activates an implemental action system at the expense of deliberative information processing [26], [27], or (3) disengages the “default mode” network as players re-engage the “task positive” network to resume gameplay [28]. Interestingly, gameplay onset was transiently associated with increased activity in sub-regions of the medial pre-frontal cortex (Figure 3) – a pattern opposite to the deactivation observed over the broader medial prefrontal region during extended periods of interactive gameplay (i.e., Figure 2). Future studies will be required to determine the replicability of these biphasic responses in the prefrontal cortex and determine their functional significance in the context of IDG play.

This study also has a number of other limitations that will need to be resolved by future research. One limitation involves the use of an asymmetric 3∶1 randomization strategy, which creates an unbalanced sampling design. We employed asymmetric randomization to enhance the size of the active play group and thereby increase statistical power to resolve relationships between gameplay-induced neural responses and attitude-related outcomes within that group. Statistical analyses were appropriately adjusted for asymmetric sample sizes in between-group comparisons, and extensive Monte Carlo analyses have confirmed that the *t* statistics utilized here remain valid in the context of 3∶1 sample size asymmetries [29]–[31]. However, future research should utilize larger total sample sizes in order to provide sufficient statistical power for well-powered within-group analyses while permitting balanced group sizes. Another limitation involved the nature of study participants. This study examined neural responses to *Re-Mission* gameplay in healthy undergraduates in order to define the basic effects of IDG play on activity of brain regions implicated in general reward-related processes. However, *Re-Mission* was specifically developed as an intervention for adolescents and young adults with cancer, and future studies will be required to determine what additional neural circuits might be engaged in players with a more deeply personal relationship to cancer. Future studies comparing gameplay responses in healthy individuals and cancer patients will also be required to determine whether *Re-Mission's* effects on out-of-game behavior stem primarily from the types of general responses observed here (e.g., coupling of cancer-related symbolic content with IDG-related activation of reward circuitry) or from cancer patient-specific responses (or perhaps from a mix of both). However, the observed association between parahippocampal response to gameplay onset and subsequently measured chemotherapy-related attitudes suggests that this game's general coupling of interactivity-induced activation of reward-related motivational circuits with cancer-related symbolic content might potentially contribute to at least some cancer-related outcomes (i.e., some effects occur among those with no direct personal experience of cancer). Nevertheless, future studies will need to analyze these attitude-related neural dynamics in cancer patients to more directly define the mechanisms by which the *Re-Mission* IDG influences cancer-related behaviors such as treatment adherence.

Given that the present data indicate a key role for interactivity in driving IDG activation of mesolimbic projection areas, it is worth considering which specific aspect of interactive play is responsible for such engagement. We hypothesize that the distinctive activation of anticipatory reward-related brain structures during IDG play is driven primarily by the players' recognition that their personal evaluative outcomes in the game (e.g., performance, self-view) depend on their instrumental actions. In other words, mesolimbic projection areas can modulate perception, learning, memory, and other cognitive functions that are engaged as part of the players' goal-directed efforts to succeed in game performance. Other psychological processes that are mobilized as a consequence of self-engagement may also contribute to the profile of neural recruitment observed during interactive gameplay. For example, the observed activations in the supplementary motor area and precuneus are consistent with previous studies implicating those structures in self-related processes [32]–[35] and behavior change [36], [37]. Data linking the activation of those structures to positive behavior change [36], [37] are consistent with the present findings in suggesting that self-engagement may play a role in mediating the effects of IDG-based behavioral interventions. However, other psychological dynamics that are correlated with the activation of reward- and self-related neural structures in this study (e.g., sensory effects on experiences of flow or virtual presence [11], [38], [39], motoric output involved in gameplay) might contribute as well. Future studies that dissociate the instrumental or mechanical components of participatory control (e.g., sensory, planning, and motoric) from its affective and motivational components (e.g., self-view, goal achievement) could deepen our understanding of the neurobiological basis for play's distinctive power in driving the development of skills, knowledge, and complex behavior [1], [3]–[6].

## Materials and Methods

### Subjects

Data were collected from 65 young adults who were recruited through posted advertisements in the Stanford University environment and screened for typical FMRI exclusions (e.g., metal in the body, psychiatric and/or cardiac drugs). Data from 8 subjects were excluded due to technical difficulties (n = 1 in the active play and n = 2 in the passive exposure group) or excessive head motion (n = 5 showing >2 mm from one acquisition of brain volume data to the next; all in the active play group), leaving a total of 57 valid subjects for analysis. Of these, 43 were randomly assigned to the “active play” group, while the remaining 14 were assigned to the “passive exposure” group. Subjects were randomized to active play vs. passive exposure groups at a 3∶1 ratio to ensure sufficient statistical power for between-group contrasts while maximizing statistical power to detect within-group relationships between gameplay events and neural responses in the active play group. Subjects were young adults (mean age = 25.3 years, SD = 9.4, range 18–50), approximately balanced in terms of sex (48% female, 52% male) and ethnically heterogeneous (52% Caucasian, 25% Asian, 10% Hispanic, 5% African American, 5% Pacific Islander, 3% Mixed).No group matching was attempted, and as expected under random assignment, the resulting groups did not differ in age, gender, or ethnic distribution (all differences p>.20). Questionnaires administered immediately after playing or watching the video game included items measuring subjects' attitudes towards cancer and chemotherapy using seven point Likert scales (e.g., “How vital is chemotherapy in the treatment of cancer?”). Subjects were contacted again, without forewarning, one month after the FMRI scanning session to complete the same attitude measures. All recruitment and research procedures were approved by the Stanford University Institutional Review Board. All subjects provided written informed consent and participated in a 1.5 hour experimental session for a flat fee of $20 per hour.

### Task

In the *Re-Mission* IDG, players pilot a miniature “nanobot” through a fictional cancer patient's body to battle cancer cells [8]. In the active play group, subjects (n = 43) played Level 1 of *Re-Mission* which involves navigating the nanobot through a string of lymph nodes while destroying lymphoma cell “enemies” with a chemotherapy gun (“chemoblaster”) (Supporting Information Video 1). Subjects received an unlimited amount of chemotherapy ammunition (and thus did not need to recharge), and were conferred invulnerability to damage received from collisions with environment walls or cancer cells, so that all could play for an equal length of time. Output generated by game software produced a two-second resolution temporal record of game events (e.g., chemoblaster shots fired, enemy cancer cells killed, damage received from enemies, environment collisions, etc.). Subjects played the videogame using a FMRI-compatible joystick (Mag Design and Engineering, Sunnyvale, CA), a video screen, and audio headphones.

In the active play group, subjects played *Re-Mission* for seven bouts of 60 sec separated by six pauses of variable duration (10–30 sec), such that game play onset was not predictable (see Figure 4). Subjects were told that they would play and rest at varying intervals throughout the scan, but were not told when or for how long. Audio-visual output from the game was recorded (Fraps, Beepa Pty Ltd) and stored for subsequent display to passive exposure group subjects. In the passive exposure group, subjects watched and listened to an audiovisual recording of the interactive gameplay event stream generated by a randomly selected previous active play group subject.

### Functional Magnetic Resonance Imaging Acquisition and Analysis

Functional images were acquired with a 1.5-Tesla General Electric MRI scanner using a standard quadrature head coil. Twenty-four contiguous axial 4-mm-thick slices (in-plane resolution 3.75×3.75 mm) with a 24 cm field of view were acquired in axial sequence from the mid-pons to the top of the skull. Functional scans were acquired with a T2*-sensitive spiral in/out pulse sequence (repetition time = 2 s, echo time = 40 ms, flip = 90 degrees). High-resolution structural scans were acquired with a T1-weighted spoiled grass sequence (repetition time = 100 ms, echo time = 7 ms, flip = 90 degrees) for localization and coregistration. Analyses were conducted with AFNI software [40]. For preprocessing, data were sinc-interpolated to correct for nonsimultaneous slice acquisition, corrected for three-dimensional motion, high-pass filtered to remove slow trends (>0.01 Hz), and normalized to percent signal change relative to the voxel mean across the entire experimental period. Inspection of motion correction estimates confirmed that none of the 57 valid subjects retained for analysis showed more than 2 mm of head movement in any dimension from one volume acquisition to the next. Initial whole-brain analyses used multiple regression to assess relationship between neural activation and regressors of interest constructed for each subject based on the temporal record of game events (binned into 2 sec intervals). These regressors included: (1) Game play vs nonplay; (2) Game play onset (2 sec); (3) Game play offset (2 sec); (4) Chemoblaster shots fired; (5) Wall collisions; (6) Enemy kills; (7) Anticipated enemy kill (2 sec prior to an enemy kill); (8) “Damage” received from cancer cells; (9) Anticipated “damage” (2 sec prior to receiving damage). Prior to entry into the regression model, regressors were convolved with a standard model of the hemodynamic response function [41]. Regressors of noninterest modeling head motion (n = 6; in plane translational and rotational motion), linear, and quadratic trends were also entered into the regression model as covariates. Whole brain regression coefficients for active play and passive exposure groups were coregistered with structural maps, spatially normalized by manually warping to Talaraich space, spatially smoothed to minimize effects of anatomic variability (FWHM = 4 mm), and submitted to single-sample t-tests (versus the hypothesis of no activation). Whole brain omnibus significance thresholds were estimated at p<.001 uncorrected using 1000 Monte Carlo simulations using AFNI's AlphSim program (or p<.05 corrected; 3×3×3.75 mm voxels; 4 mm FWHM; cluster threshold = 4 voxels). Regression coefficients for active play versus passive exposure groups were compared across groups using a two-sample t-test (corrected for unbalanced group size).

A priori volume of interest (VOI) analyses were applied to test primary hypotheses regarding group differences in the activation of reward-related regions within 2 sec following gameplay onset. VOI significance values were estimated over 4 averaged volumes specified as 6 mm diameter spheres centered bilaterally on ventral striatal regions implicated in reward anticipation (i.e., nucleus accumbens (Right/Anterior/Superior RAS = ±11,11,−2) and putamen (±17,12,0)) in previous research [42]. Overall statistical significance of primary hypothesis tests was based on Bonferroni-corrected significance threshold of p<.025 for each of the two primary bilateral tests (total family-wise p<.05). Secondary analyses of each of the 4 individual foci tested were conducted at Bonferroni-corrected significance threshold of .0125 (family-wise p<.05).

Because reward anticipation activates mesolimbic projection regions [16], [17], we predicted that the active play group would show increases in: (1) activation of sensorimotor circuits (i.e., primary sensory and motor cortices), perceptual circuits (i.e., visual cortex) and arousal-related structures (i.e., thalamus) for gameplay versus rest (nonplay); (2) activation of reward-related circuits (i.e., mesolimbic projection regions including the caudate, putamen, and nucleus accumbens) in response to the onset of game play; and (3) activation of circuits associated with interruption and aversion (i.e., anterior cingulate, anterior insula) in response to the offset of game play. Based on the hypothesis that interactive engagement in play engages motivation-related brain circuits, we additionally predicted that sensorimotor-, arousal-, reward-, memory-, and interruption/aversion-related activation dynamics would be significantly more pronounced for subjects in the active play group than in the passive exposure group.

### Attitude measurement and analysis

To identify potential “in-game” neural response correlates of post-play attitudes toward chemotherapy, regression-based exploratory analyses assessed the relationship between individual differences in chemotherapy-related attitudes and individual differences in peak impulse responses following the initiation/resumption of gameplay. Both immediately after the gameplay session and one month later at an unannounced follow-up, subjects were asked to evaluate on a 7-point scale, “How important is chemotherapy in the treatment of cancer?” and “How vital is chemotherapy in the treatment of cancer?” Responses were averaged at each assessment point to provide a composite measure of chemotherapy-related attitude (Cronbach's alpha = .91). Initial whole-brain analyses examined empirical correlates of chemotherapy-related attitudes in neural responses to gameplay onset. Because these were exploratory analyses targeting small subcortical regions, we utilized the same omnibus stringency threshold (p<.001) but a smaller cluster threshold (k = 3) to identify potential activation foci. To plot the association between individual differences in peak neural response to gameplay onset and attitudes, a VOI-based analysis extracted individual average values of peak phasic response to gameplay onset/resumption (empirically determined to occur 6 sec after play onset) with a 6 mm diameter focus centered over the region of parahippocampal cortex found to show strongest association with post-play attitudes in initial whole-brain analyses. The magnitude of linear association was summarized by both Pearson correlation and outlier-robust Spearman correlation.

## Supporting Information

Table S1 **Play versus rest activation foci.** Significant activation foci defined by Talairach-Tournoux Atlas coordinates expressed as R = Right to Left; A = Anterior to Posterior, S = Superior to Inferior.
(DOCX)

Table S2 **Game onset activation foci.** Significant activation foci defined by Talairach-Tournoux Atlas coordinates expressed as R = Right to Left; A = Anterior to Posterior, S = Superior to Inferior.
(DOCX)

Table S3 **Game offset activation foci.** Significant activation foci defined by Talairach-Tournoux Atlas coordinates expressed as R = Right to Left; A = Anterior to Posterior, S = Superior to Inferior.
(DOCX)

Table S4 **Activation foci associated with chemoblaster shots fired.** Significant activation foci defined by Talairach-Tournoux Atlas coordinates expressed as R = Right to Left; A = Anterior to Posterior, S = Superior to Inferior. Active group n = 43.
(DOCX)

Table S5 **Activation foci associated with enemy cancer cell killed.** Significant activation foci defined by Talairach-Tournoux Atlas coordinates expressed as R = Right to Left; A = Anterior to Posterior, S = Superior to Inferior. Active group n = 43.
(DOCX)

Table S6 **Game onset activation foci associated with post-play attitudes toward chemotherapy within the active play group.** Significant activation foci (p<.001, uncorrected, minimum k = 3 voxels) defined by Talairach-Tournoux Atlas coordinates expressed as R = Right to Left; A = Anterior to Posterior, S = Superior to Inferior.
(DOCX)
