# Supplementary material for: Interactivity and Reward-Related Neural Activation during a Serious Videogame
Source: PLoS One. 2012 Mar 19;7(3):e33909. doi: 10.1371/journal.pone.0033909 (PMC3307771; doi:10.1371/journal.pone.0033909)
Supplement: Table S1 — Play versus rest activation foci. Significant activation foci defined by Talairach-Tournoux Atlas coordinates expressed as R = Right to Left; A = Anterior to Posterior, S = Superior to Inferior. (DOCX) [file pone.0033909.s001.docx]

**Table S1. Play versus rest activation foci.** Significant activation foci defined by Talairach-Tournoux Atlas coordinates expressed as R = Right to Left; A = Anterior to Posterior, S = Superior to Inferior.

**1a.** Active group

| **Talairach-Tournoux Atlas Region** | **Peak Z-Score** | **R** | **A** | **S** | **Cluster size** |
| --- | --- | --- | --- | --- | --- |
|  |  |  |  |  |  |
| Right Superior Frontal Gyrus | -4.02 | 15 | 60 | 12 | 5 |
| Left Middle Frontal Gyrus | 5.35 | -41 | 49 | 19 | 74 |
| Right Inferior Frontal Gyrus | -4.60 | 45 | 30 | -7 | 22 |
| Left Subcallosal Gyrus | -4.14 | -26 | 8 | -14 | 4 |
| Right Parahippocampal Gyrus | -4.02 | 30 | 4 | -14 | 11 |
| Right Middle Temporal Gyrus | -4.90 | 64 | -15 | -3 | 64 |
| Left Middle Temporal Gyrus | -6.92 | -56 | -38 | 4 | 107 |
| Right Culmen | 13.00 | 4 | -56 | -3 | 10848 |

**1b.** Passive group

| **Talairach-Tournoux Atlas Region** | **Peak Z-Score** | **R** | **A** | **S** | **Cluster size** |
| --- | --- | --- | --- | --- | --- |
|  |  |  |  |  |  |
| Left Middle Frontal Gyrus | 3.45 | -34 | 4 | 49 | 4 |
| Right Precentral Gyrus | -3.93 | 52 | 0 | 12 | 10 |
| Right Lentiform Nucleus | -3.90 | 22 | -4 | -3 | 5 |
| Right Inferior Parietal Lobule | -3.92 | 52 | -41 | 49 | 5 |
| Right Precuneus | 5.11 | 11 | -45 | 53 | 61 |
| Left Precuneus | 4.15 | -26 | -49 | 53 | 42 |
| Left Middle Occipital Gyrus | 5.83 | -41 | -75 | 8 | 1023 |

**1c.** Active group > Passive group

| **Talairach-Tournoux Atlas Region** | **Peak Z-Score** | **R** | **A** | **S** | **Cluster size** |
| --- | --- | --- | --- | --- | --- |
| Left Medial Frontal Gyrus | -4.39 | 0 | 56 | -7 | 27 |
| Left Superior Frontal Gyrus | -4.00 | -15 | 49 | 23 | 35 |
| Left Medial Frontal Gyrus | -4.70 | -15 | 34 | 42 | 10 |
| Right Middle Frontal Gyrus | 5.25 | 34 | 30 | 27 | 34 |
| Left Inferior Frontal Gyrus | -4.99 | -34 | 30 | -11 | 19 |
| Left Inferior Frontal Gyrus | -3.56 | -49 | 26 | 1 | 4 |
| Right Insula | 5.56 | 30 | 19 | 4 | 298 |
| Left Superior Frontal Gyrus | 6.59 | 0 | 4 | 49 | 1199 |
| Left Insula | 3.66 | -41 | 0 | 8 | 8 |
| Left Parahippocampal Gyrus | -4.28 | -22 | -15 | -11 | 6 |
| Right Cingulate Gyrus | 3.76 | 8 | -26 | 42 | 4 |
| Left Middle Temporal Gyrus | -3.81 | -60 | -30 | 1 | 4 |
| Left Supramarginal Gyrus | -5.23 | -41 | -52 | 34 | 98 |
| Left Precuneus | -4.74 | -4 | -52 | 34 | 46 |
| Right Inferior Parietal Lobule | -4.18 | 49 | -52 | 46 | 10 |
| Right Culmen | 5.79 | 4 | -56 | -3 | 96 |
| Left Precuneus | 5.09 | -11 | -60 | 49 | 32 |
| Left Precuneus | 4.49 | -19 | -71 | 34 | 8 |
| Left Middle Occipital Gyrus | 3.96 | -26 | -82 | 19 | 5 |
