# Supplementary material for: Interactivity and Reward-Related Neural Activation during a Serious Videogame
Source: PLoS One. 2012 Mar 19;7(3):e33909. doi: 10.1371/journal.pone.0033909 (PMC3307771; doi:10.1371/journal.pone.0033909)
Supplement: Table S2 — Game onset activation foci. Significant activation foci defined by Talairach-Tournoux Atlas coordinates expressed as R = Right to Left; A = Anterior to Posterior, S = Superior to Inferior. (DOCX) [file pone.0033909.s002.docx]

**Table S2. Game onset activation foci.** Significant activation foci defined by Talairach-Tournoux Atlas coordinates expressed as R = Right to Left; A = Anterior to Posterior, S = Superior to Inferior.“ indicates additional element of an extended activation cluster of size listed above.

**2a.** Active group

| **Talairach-Tournoux Atlas Region** | **Peak Z-Score** | **R** | **A** | **S** | **Cluster size** |
| --- | --- | --- | --- | --- | --- |
| Left Superior Frontal Gyrus | -4.13 | 0 | 56 | -22 | 12 |
| Left Middle Frontal Gyrus | -4.17 | -41 | 49 | -3 | 24 |
| Right Superior Frontal Gyrus | -4.61 | 34 | 49 | 16 | 4 |
| Right Medial Frontal Gyrus | 3.77 | 0 | 45 | 42 | 5 |
| Right Medial Frontal Gyrus | 6.68 | 8 | 41 | -11 | 726 |
| Left Nucleus Accumbens | 6.10 | -11 | 14 | -6 | " |
| Left Putamen | 6.34 | -17 | 10 | -6 | " |
| Right Putamen | 6.67 | 18 | 9 | -7 | " |
| Right Middle Frontal Gyrus | -4.09 | 30 | 34 | 23 | 19 |
| Left Claustrum | 4.11 | -22 | 26 | 4 | 10 |
| Left Superior Temporal Gyrus | 4.25 | -34 | 15 | -33 | 4 |
| Right Precentral Gyrus | 4.26 | 56 | 0 | 23 | 12 |
| Right Middle Temporal Gyrus | 4.89 | 45 | 0 | -22 | 11 |
| Left Uncus | 4.62 | -22 | -4 | -29 | 10 |
| Left Postcentral Gyrus | 3.69 | -56 | -8 | 16 | 18 |
| Right Middle Temporal Gyrus | 3.95 | 45 | -8 | -14 | 5 |
| Left Cingulate Gyrus | 4.44 | -4 | -11 | 31 | 6 |
| Left Thalamus | 3.98 | -8 | -11 | -3 | 4 |
| Right Posterior Insula | 4.79 | 41 | -15 | 19 | 24 |
| Left Posterior Insula | 4.48 | -38 | -15 | 19 | 15 |
| Right Parahippocampal Gyrus | 4.16 | 26 | -15 | -14 | 4 |
| Left Precentral Gyrus | 4.05 | -30 | -19 | 57 | 4 |
| Right Superior Temporal Gyrus | 4.78 | 52 | -22 | 1 | 32 |
| Left Thalamus | 4.89 | -4 | -22 | 1 | 8 |
| Left Culmen | -4.87 | -15 | -26 | -22 | 12 |
| Right Culmen | -4.49 | 26 | -30 | -22 | 13 |
| Left Posterior Cingulate Gyrus | 3.83 | -4 | -41 | 38 | 4 |
| Left Precuneus | -6.62 | 0 | -49 | 61 | 1980 |
| Right Middle Occipital Gyrus | -4.02 | 19 | -94 | 16 | 9 |

**2b.** Passive group

| **Talairach-Tournoux Atlas Region** | **Peak Z-Score** | **R** | **A** | **S** | **Cluster size** |
| --- | --- | --- | --- | --- | --- |
| Left Superior Frontal Gyrus | 3.99 | -26 | 41 | 16 | 11 |
| Left Cingulate Gyrus | 3.75 | 0 | 30 | 27 | 9 |
| Right Cingulate Gyrus | 4.29 | 4 | 15 | 31 | 20 |
| Left Insula | 3.87 | -30 | 15 | 8 | 9 |
| Right Insula | 3.94 | 34 | 15 | 8 | 7 |
| Left Postcentral Gyrus | 4.15 | -52 | -15 | 16 | 11 |
| Left Claustrum | 3.82 | -38 | -15 | -3 | 5 |
| Left Cingulate Gyrus | 4.69 | -4 | -19 | 27 | 10 |
| Right Paracentral Lobule | 3.6 | 4 | -26 | 46 | 5 |
| Right Parahippocampal Gyrus | 4.38 | 22 | -49 | 8 | 13 |
| Right Postcentral Gyrus | -4.12 | 11 | -49 | 64 | 8 |
| Right Inferior Temporal Gyrus | -4.55 | 49 | -64 | 1 | 12 |
| Left Middle Temporal Gyrus | -3.96 | -45 | -64 | 8 | 11 |
| Left Precuneus | 4.35 | -15 | -68 | 19 | 5 |
| Right Precuneus | -4.09 | 15 | -71 | 49 | 10 |
| Right Cuneus | -4.43 | 26 | -75 | 12 | 5 |

**2c.** Active group > Passive group

| **Talairach-Tournoux Atlas Region** | **Peak Z-Score** | **R** | **A** | **S** | **Cluster size** |
| --- | --- | --- | --- | --- | --- |
| Right Superior Frontal Gyrus | -5.06 | 34 | 49 | 16 | 18 |
| Right Middle Frontal Gyrus | -4.16 | 30 | 34 | 23 | 12 |
| Right Insula | -3.66 | 34 | 15 | 8 | 4 |
| Right Medial Frontal Gyrus | -4.51 | 11 | 15 | 46 | 4 |
| Right Cingulate Gyrus | -3.77 | 11 | -26 | 42 | 4 |
| Left Caudate | -3.85 | -19 | -26 | 19 | 4 |
| Right Inferior Parietal Lobule | -4.83 | 34 | -38 | 38 | 14 |
| Left Inferior Parietal Lobule | -3.62 | -30 | -38 | 38 | 6 |
| Left Inferior Parietal Lobule | -4.20 | -45 | -38 | 53 | 5 |
| Left Culmen | -3.80 | 0 | -49 | -7 | 5 |
|  |  |  |  |  |  |
