# Supplementary material for: Interactivity and Reward-Related Neural Activation during a Serious Videogame
Source: PLoS One. 2012 Mar 19;7(3):e33909. doi: 10.1371/journal.pone.0033909 (PMC3307771; doi:10.1371/journal.pone.0033909)
Supplement: Table S3 — Game offset activation foci. Significant activation foci defined by Talairach-Tournoux Atlas coordinates expressed as R = Right to Left; A = Anterior to Posterior, S = Superior to Inferior. (DOCX) [file pone.0033909.s003.docx]

**Table S3. Game offset activation foci.** Significant activation foci defined by Talairach-Tournoux Atlas coordinates expressed as R = Right to Left; A = Anterior to Posterior, S = Superior to Inferior.“ indicates additional element of an extended activation cluster of size listed above.

**3a.** Active group

| **Talairach-Tournoux Atlas Region** | **Peak Z-Score** | **R** | **A** | **S** | **Cluster size** |
| --- | --- | --- | --- | --- | --- |
| Right Medial Frontal Gyrus | 4.10 | 4 | 71 | -3 | 5 |
| Left Superior Frontal Gyrus | 4.28 | -8 | 64 | -7 | 20 |
| Left Orbitofrontal Cortex | -6.30 | -8 | 26 | -11 | 70 |
| Left Caudate | -3.90 | -15 | 22 | 16 | 5 |
| Left Precentral Gyrus | 4.28 | -60 | 11 | 8 | 9 |
| Right Putamen | -4.02 | 19 | 8 | -3 | 5 |
| Left Inferior Parietal Lobule | -4.72 | -41 | -64 | 46 | 39 |
| Left Middle Occipital Gyrus | 7.99 | -45 | -71 | 8 | 11803 |
| Right Anterior Cingulate | 6.60 | 6 | 13 | 36 | " |
| Right Anterior Insula | 7.11 | 31 | 18 | 7 | " |
| Left Anterior Insula | 5.65 | -33 | 16 | 7 | " |

**3b.**Passive group

| **Talairach-Tournoux Atlas Region** | **Peak Z-Score** | **R** | **A** | **S** | **Cluster size** |
| --- | --- | --- | --- | --- | --- |
| Left Middle Frontal Gyrus | -4.51 | -41 | 34 | 19 | 4 |
| Right Inferior Frontal Gyrus | 3.82 | 38 | 26 | 12 | 6 |
| Right Precentral Gyrus | 3.96 | 38 | 15 | 34 | 23 |
| Left Postcentral Gyrus | -3.93 | -56 | -8 | 16 | 6 |
| Right Thalamus | 3.96 | 11 | -19 | 12 | 8 |
| Right Superior Temporal Gyrus | 4.42 | 52 | -45 | 19 | 17 |
| Left Lingual Gyrus | 4.04 | -22 | -49 | 1 | 31 |
| Left Superior Temporal Gyrus | 4.06 | -56 | -52 | 16 | 7 |
| Right Postcentral Gyrus | 4.25 | 4 | -52 | 64 | 6 |
| Right Precuneus | 4.15 | 11 | -56 | 27 | 22 |
| Right Middle Temporal Gyrus | 3.75 | 45 | -64 | 16 | 10 |
| Right Precuneus | 4.87 | 4 | -68 | 19 | 317 |
| Left Middle Occipital Gyrus | 4.54 | -38 | -79 | 12 | 17 |
|  |  |  |  |  |  |

**3c .** Active group > Passive group

| **Talairach-Tournoux Atlas Region** | **Peak Z-Score** | **R** | **A** | **S** | **Cluster size** |
| --- | --- | --- | --- | --- | --- |
| Right Superior Frontal Gyrus | 4.58 | 26 | 38 | 23 | 19 |
| Left Middle Frontal Gyrus | 4.18 | -41 | 38 | 16 | 9 |
| Right Insula | 4.90 | 38 | 8 | 4 | 200 |
| Left Inferior Frontal Gyrus | 4.28 | -49 | 4 | 23 | 7 |
| Right Middle Frontal Gyrus | 5.26 | 26 | -4 | 57 | 856 |
| Left Insula | 5.21 | -38 | -4 | 8 | 66 |
| Left Postcentral Gyrus | 4.50 | -56 | -19 | 27 | 21 |
| Left Thalamus | 4.29 | -8 | -19 | 4 | 13 |
| Left Insula | 3.85 | -34 | -19 | 12 | 5 |
| Right Red Nucleus | 3.58 | 4 | -19 | -14 | 4 |
| Right Parahippocampal Gyrus | 3.44 | 26 | -19 | -11 | 4 |
| Right Thalamus | 4.40 | 15 | -22 | 4 | 13 |
| Right Inferior Parietal Lobule | 3.85 | 56 | -41 | 27 | 7 |
| Left Culmen | 5.39 | -11 | -49 | -11 | 101 |
| Right Precuneus | 3.58 | 26 | -75 | 46 | 4 |
