# Supplementary material for: Interactivity and Reward-Related Neural Activation during a Serious Videogame
Source: PLoS One. 2012 Mar 19;7(3):e33909. doi: 10.1371/journal.pone.0033909 (PMC3307771; doi:10.1371/journal.pone.0033909)
Supplement: Table S5 — Activation foci associated with enemy cancer cell killed. Significant activation foci defined by Talairach-Tournoux Atlas coordinates expressed as R = Right to Left; A = Anterior to Posterior, S = Superior to Inferior. Active group n = 43. (DOCX) [file pone.0033909.s005.docx]

**Table S5 - Activation foci associated with enemy cancer cell killed.**

Significant activation foci defined by Talairach-Tournoux Atlas coordinates expressed as R = Right to Left; A = Anterior to Posterior, S = Superior to Inferior. Active group n = 43.
