# Supplementary material for: Interactivity and Reward-Related Neural Activation during a Serious Videogame
Source: PLoS One. 2012 Mar 19;7(3):e33909. doi: 10.1371/journal.pone.0033909 (PMC3307771; doi:10.1371/journal.pone.0033909)
Supplement: Table S6 — Game onset activation foci associated with post-play attitudes toward chemotherapy within the active play group. Significant activation foci (p<.001, uncorrected, minimum k = 3 voxels) defined by Talairach-Tournoux Atlas coordinates expressed as R = Right to Left; A = Anterior to Posterior, S = Superior to Inferior. (DOCX) [file pone.0033909.s006.docx]

**Table S6. Game onset activation foci associated with post-play attitudes toward chemotherapy within the active play group.** Significant activation foci (p < .001, uncorrected, minimum k=3 voxels) defined by Talairach-Tournoux Atlas coordinates expressed as R = Right to Left; A = Anterior to Posterior, S = Superior to Inferior.

**6a. Attitudes assessed immediately post gameplay.**

| **Talairach-Tournoux Atlas Region** | **Peak Z Score** | **R** | **A** | **S** | **Cluster size** |
| --- | --- | --- | --- | --- | --- |
|  |  |  |  |  |  |
| Bilateral Medial Frontal Gyrus / MPFC | 3.54 | 0 | 63 | -4 | 8 |
| Left Anterior Cingulate | 3.20 | -3 | 37 | 12 | 4 |
| Right Medial Frontal Gyrus | 3.36 | 3 | 28 | -11 | 5 |
| Right Middle Temporal Gyrus | 3.43 | 46 | -4 | -19 | 10 |
| Left Parahippocampal Gyrus | 3.42 | -26 | -15 | -19 | 3 |
| Left Inferior Temporal Gyrus, Left BA 20 | 3.47 | -53 | -28 | -18 | 3 |

**6b. Attitudes assessed one month post gameplay.**

| **Talairach-Tournoux Atlas Region** | **Peak Z Score** | **R** | **A** | **S** | **Cluster size** |
| --- | --- | --- | --- | --- | --- |
|  |  |  |  |  |  |
| Left Inferior Frontal Gyrus | 3.41 | 60 | 11 | 15 | 3 |
| Left Parahippocampal Gyrus | 3.49 | -33 | -7 | -20 | 8 |
|  |  |  |  |  |  |
|  |  |  |  |  |  |
